# *Cornus Mas* L improves Antioxidant Status in the Liver, Lung, Kidney, Testis and Brain of Ehrlich Ascites Tumor Bearing Mice

**DOI:** 10.31557/APJCP.2020.21.9.2531

**Published:** 2020-09

**Authors:** Seher Yılmaz, Ayşe Yeşim Göçmen, Ersin Karataş, Adem Tokpınar

**Affiliations:** 1 *Department of Anatomy, Yozgat Bozok University Faculty of Medicine, Yozgat, Turkey. *; 2 *Department of Biochemistry, Yozgat Bozok University Faculty of Medicine, Yozgat, Turkey. *; 3 *Department of Molecular Biology and Genetics, Gebze Technical University, Kocaeli Turkey. *

**Keywords:** Cornus Mas L, mice, tumor, antioxidant

## Abstract

Cornelian Cherry (*Cornus Mas* L) has widespread use due to its anti-inflammatory, anti-carcinogenic and anti-oxidant properties. In this study, the effects of *Cornus Mas* L (*C. mas* L) in different dosages on the biochemical values of mice organs were investigated in the Ehrlich Ascites tumor model, which originated from mice breast adenocarcinoma and developed in Balb/C mice. In our study, 32 Balb/C type male mice were used. Ehrlich Ascites Tumor (EAT) cells (1x10^6^ EAT cell) from the stock animal were injected into all the mice in an intraperitoneal way. Experimental groups were given 100 and 200mg/kg *C. mas* L extract intraperitoneally for 9 days. The weights of the animals were recorded every day and were sacrificed on the 9^th^ day. To estimate tumor proliferation of the lung, brain, kidney, liver, and testis, antioxidant parameters were recorded including, the reduced glutathione (GSH), lipid peroxidation, glutathione S-transferase (GST), superoxide dismutase (SOD) and catalase (CAT). Treatments of different doses of *C. mas* L. meaningfully (p < 0.05) modulated the lung, brain, kidney, liver and testis tissues antioxidant parameters as compared to the control. Our study showed the anti-tumor effect of *C. mas* L. in assisted tumor development with EAT cells, conceivably moderated by the enhancement of oxidative stress due to numerous mechanisms.

## Introduction

New developments in cancer treatment are constantly being made. Recently, the use of plant extracts in cancer treatment is increasing. One of these herbal extracts is *C. mas *L (Weiss et al., 1981).

*C. mas* L, is a medicinal plant that has a high level of antioxidant activity. *C. mas* L. is a plant that has high nutritional value and also has therapeutic properties. It grows in Asia and Europe. Especially in Turkey, it is grown in gardens for its fruit and it grows wild in northern Anatolian forests. (Kayaalp., 1996). There are numerous articles in the literature that demonstrate the antineoplastic mechanism of curcumin. *C. Mas* L is known to increase the rate of death of some types of cancer cells and stop the division of Tumor Cells (Peyman et al., 2014, Turhan. 1999). Earlier studies indicated that *C. Mas* L was a potent antioxidant and had an anticancerogenic effect. (Savikin et al., 2009).

Anti-tumoral effects of plant extracts have been tested on several cancer models, one of which is the EAT model (Folkman et al., 1992, Ozaslan et al., 2011). The EAT model first appeared as a spontaneous breast adenocarcinoma in a female mouse. Tumor pieces were transplanted subcutaneously into mice and transformed into experimental tumors. Then another tumor form was obtained growing in liquid form in the peritoneum of the mice. This model has been included in many studies (Folkman et al., 1992, Lazebnik et al., 1991, Ozaslan et al., 2011, Siems et al., 1993). The cytotoxic effect of *C. mas *L. on several cancer cell lines mainly focused on its flower and leave extracts. The operational definition of Oxidative stress is an increase in oxidants and / or a decrease in antioxidants. Subsequently, to assess the overall or net oxidative status in the body, the total levels of oxidants and antioxidants should be measured concurrently. Oxidative stress has been involved in the pathogenesis of cancer (Feng et al., 2012) and might impair tissue injuries in patients with cancer (Wu et al., 2017).

A higher lipid peroxidation and disturbed antioxidant enzyme activities (superoxide dismutase (SOD), catalase (CAT), glutathione peroxidase (GPx) as well as glutathione (GSH) are generally detected in cancer patients (Feng et al., 2012). 

With the aim of estimating the overall antioxidative status, the total antioxidant status (TAS) is often measured. Similarly, total oxidant status (TOS) is used to quantify the overall state Balbto measure the net oxidative stress in the body (Feng et al., 2012). In the current study, we postulated that there is a possible remote effect of tumor preconditioning on tissue oxidant stress status.

Previous researchers found various phytochemical and pharmacological studies on *C. mas* L. fruits, seeds and other parts in experimental animal models (Tomás et al., 2013, Zarei et al., 2014). Due to there not being any experimental studies on antitumor activity of *C. mas* L. fruits on the lung, brain, kidney, liver and testis tissues we saw a necessity to assess the *C. mas* L. extract for potential antitumor effects and influence on antioxidant status against mice with Balb/C Ehrlich ascites carcinoma (EAT).

As a result of regular consumption of *C. mas* L fruits among people and its use for anticancer purposes in folk medicine, the aim of this study is to investigate the antitumor effect of *C. mas* L. fruit, which has not been tested in this model on the experimental EAT ascitic tumor model in Balb/C mice.

## Materials and Methods

*Methods*

In this study, the Balb/C type mouse was used from Erciyes University Experimental Research Center The study on experimental animals was made in accordance with the decision of the Local Ethics Committee of Animal Experiments, Erciyes University, dated October 12, 2016, with the number 16/119. The evaluation of the tissues obtained after the experiment was carried out in the research laboratory of Yozgat Bozok University.

*The type, number and distribution of subjects used in the research*

In this study, male mice were used in 8-10 weekly, average 25-30 GR and 32 units of Balb/C type, with the characteristics of the animal’s ancestry, type, gender, age and weight selection in other studies.

*Experimental Procedure*

Mice were preserved in specially prepared, automatically air-conditioned rooms with a constant temperature of 21ºC and 12 hours of light/dark periods during the research. In the study, the stock mouse was first created to obtain enough EAT cells before the groups were created. The ascites fluid taken with the help of a stock animal injector was suspended in 0.1 ml PBS and counted in Thoma, and the 1x10^6^ EAT cell was injected intraperitoneal to create a liquid tumor in rats, and animal weights were weighed daily. 

The fresh fruit samples were transported to the laboratory and the seeds were removed manually. The fruits were extracted with methanol in a Soxhlet apparatus for 24 h. Following this, the evaporation of the methanol was done with a rotary evaporator. The water extracts were also arranged by adding boiling water to 20 g of material in a glass flask and incubated at room temperature for 12 hours on a rotating shaker (250 rpm). The mixture was sieved using Whatman (No.1) filter paper and then the excess was lyophilized. All of the extracts were stored in a freezer at -20°C until use. The powder of the concentrated extract was weighed each day for each animal experiment after being adjusted to 0.2 ml of PBS (Phosphate Buffer Saline) and filtered to 100 and 200 mg/kg/day of *C. mas *L. extract for experimental groups, and then injected intraperitoneally.

*Experimental Groups*

Group 1: Negative control group (n = 8): Cancer was not formed and animals were fed with a normal diet for 9 days with 0.1 ml of serum physiological (SF) administered intraperitoneally. 

Group 2: Positive control group (n = 8): The rats in this group were administered 0.1 ml of ascites fluid with a 1x106 EAT cells intraperitoneally in the abdominal region at 0 days. From the day 0 to 9 days, the rats had intraperitoneal 0.5 ml saline injections. 

Group 3: Treatment Group (100 mg) (n = 8): The rats involved in this group were administered 0.1 ml of ascites fluid with a 1x106 EAT cells intraperitoneally in the abdominal region at 0 days. For 9 days from the day 0, 100mg/kg/day *C. Mas* L was injected into the mice intraperitoneally. 

Group 4: Treatment group (200 mg) (n = 8): The rats in this group underwent injections of 0.1 ml of ascites fluid with a 1x106 EAT cells intraperitoneally in the abdominal area at day 0. For 9 days from the day 0, 200 mg/kg/day *C. Mas* L was intraperitoneally injected into the mice. 

All animals were sacrificed under general anesthesia of ketamine-xylosine (75mg/kg, 15mg/kg) on the 10th day and lung, liver, testicular, brain, kidney tissues were taken.

*Biochemical Analysis*

*Tissue Preparation*

Homogenization was conducted on all lung, brain, kidney, liver and testis tissues with a RIPA buffer containing 50 mmol/l Tris–HCl, 150 mmol/l NaCl, 5 mmol/l EDTA, pH 7.4, 1% of aprotinin, 1% of Nonidet P-40, 1% of sodium deoxycholate, 0.1% of SDS, 50 mmol/l of NaF and 0.1 mmol/l of Na3VO4; together with a proteinase inhibitor cocktail (Merck KGaA, Darmstadt, Germany). Following centrifugation (Beckman Coulter, Krefeld, Germany) for 10 min at 4◦C at a speed of 14,000 rpm, the supernatant produced was administered as the total protein. The Bradford method (Merlo et al., 2006) was used to measure protein concentrations.

*Markers for Oxidative Stress*

The oxidative stress parameters, for the total antioxidant status (TAS) and total oxidant status (TOS) levels, were determined with an automatic biochemical analyzer (c800, Abbott, USA). The tissue TAS level was measured by Erel 2004 (Erel et al., 2004). Following this, the assay relied on the ability of antioxidants in the sample to inhibit ABTS (2.2 azino-di-3-ethylbenz-thiazoline sulfonate) from being oxidized into ABTS+ by a peroxidase metmyoglobin. An antioxidant with a concentration of 1.65 mmol/l was implemented as the standard in calculating antioxidant levels. The TAS level was expressed in mmol trolox equivalent/l (mmol Trolox equiv./l). The TOS level of the tissues were measured by the Erel 2005 TOS method (Erel et al., 2005) that relied on the oxidation of ferrous ions into ferric ions in the company of various oxidative species within an acidic medium. Xylenol orange was used to measure the ferric ion concentrations. Calibration of the assay was completed with an standard hydrogen peroxide solution of 39.16 µmol/l and subesquently, the results were expressed in µmol H_2_O_2_equivalent/l (µmol H_2_O_2_equiv./l). The TOS/TAS ratio was defined as an oxidative stress index (OSI). The superoxide dismutase (SOD) levels of the tissues were obtained by the nitroblue tetrazolium reduction technique in accorance with Beauchamp (Beauchamp., 1971). The SOD measurements were reported to the quantity of protein in each sample.

*Measurement of CAT, GPx*

Activities of CAT and Se-GSH-Px and the levels of GSH and GSSG was measured in tissues by the modified methods of Ozturk et al., (2004).

*Statistical Analysis*

Data were analysed using the statistical package program SPSS for Windows^®^ 23.0 (SPSS, Chicago, IL, USA).The results are presented as the mean ±standard deviation (Mean±SD) of replicates. Data in all experiments were analyzed for statistical significance using analyses of variance (One –Way ANOVA). Post hoc analyses were made to compare parameters of different groups. The p-value <0.05 was considered as statistically significant. GraphPad Prism for Windows package program (version 7.00 La jolla California USA) was used.

## Results

**Figure 1:**
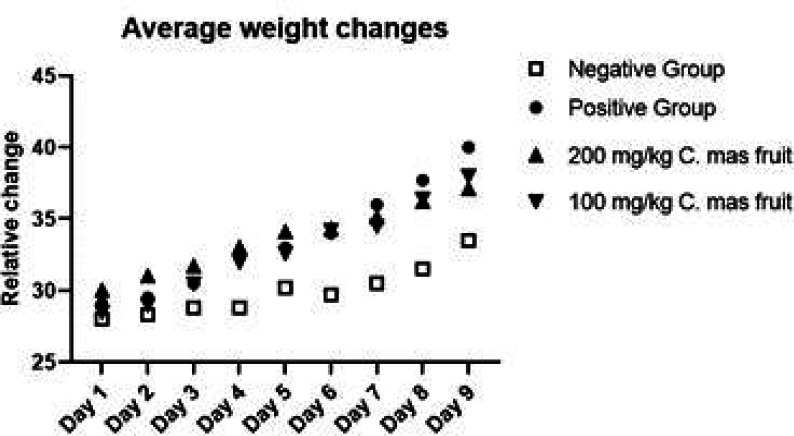
Average Weight Changes

During the experiment, a rapid increase was observed from the 5th day in the tumor control group when the data on the body weights of the animals belonging to the groups were viewed daily (Figure 1). The cause of weight gain in the animals is the increase of tumor cells in the intraperitoneal fluid. In the treatment groups, it was observed that the increase began to increase from the 6th day. The last day and first day weight difference was observed most in the tumor control group (10.9 g), followed by the treatment group (9.5 g) which was given 100 mg/kg *C. Mas* L and the group given 200 mg/kg *C. Mas *L. (7.1 g) respectively.

*Measurement of CAT, GPx*

**Figure 2:**
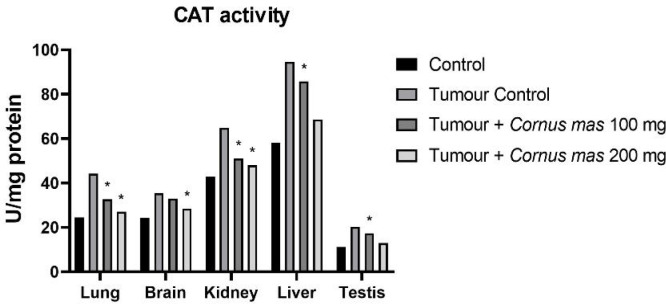
Mean CAT Activity Levels in All Groups. The data are expressed as mean ± standard deviation (*p<0.05 vs. Control group. One way ANOVA, post hoc Tukey test)

**Figure 3:**
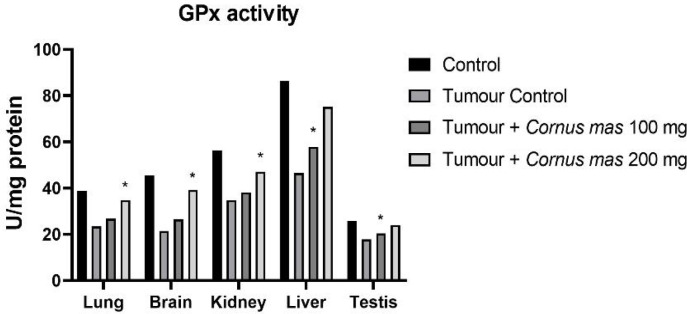
Mean GPx Activity Levels in All Groups. The data are expressed as mean ± standard deviation (*p<0.05 vs. Control group. One way ANOVA, post hoc Tukey test)

CAT activity level was measured for all lung, brain, kidney, liver and testis tissues. The tumor control group had the highest value than in all groups whereas the control group had the lowest value (Figure 2). In lung and kidney tissues, Tumor + *C. mas* L. 100 mg and Tumor + *Cornus mas* L. 200 mg groups were significantly higher overall compared with other groups (p<0.05). Likewise, in liver and testis tissues the Tumor + *C. mas* L. 100 mg groups were significantly higher overall over other groups (p<0.05). However, brain tissue in the Tumor + *C. mas* L. 200 mg group had the weightiest value. Similarly, GPx activity level was the lowest value for the Tumor control groups in all tissues and also the highest level was the Control group between all tissues (Figure 3). In the lung, brain and kidney tissues the Tumor + *C. mas* L. 100 mg group showed significant effect while in the liver and testis groups the Tumor + *C. mas* L. 200 mg groups showed important effect (p<0.05).

*Oxidative Stress Markers Result*

**Figure 4:**
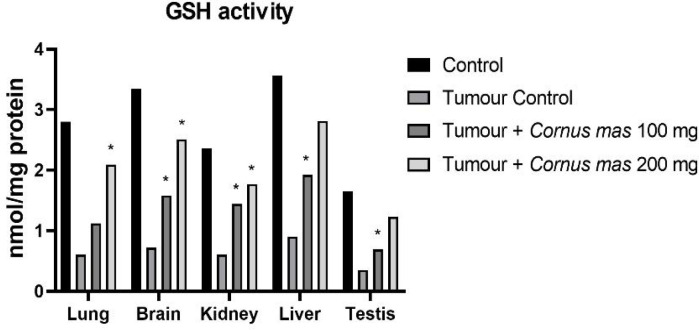
Mean GSH Activity Levels in All Groups. The data are expressed as mean ± standard deviation (*p<0.05 vs. Control group. One way ANOVA, post hoc Tukey test)

**Figure 5:**
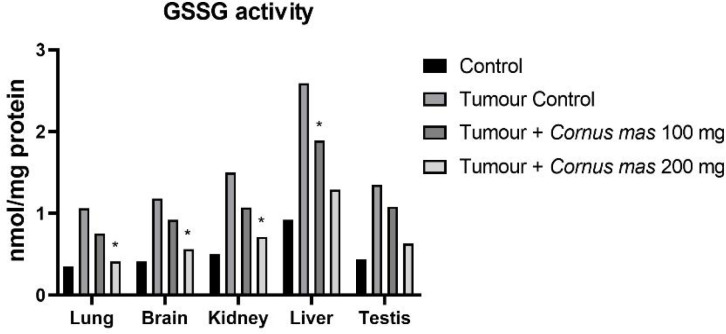
Mean GSSG Activity Levels in All Groups. The data are expressed as mean ± standard deviation (*p<0.05 vs. Control group. One way ANOVA, post hoc Tukey test)

**Figure 6:**
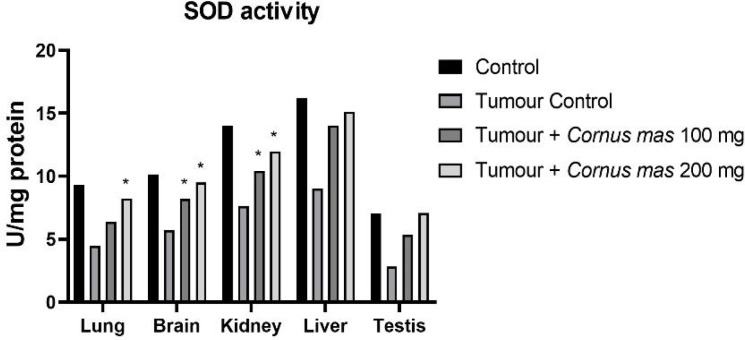
Mean SOD Activity Levels in All Groups. The data are expressed as mean ± standard deviation (*p<0.05 vs. Control group. One way ANOVA, post hoc Tukey test)

**Figure 7:**
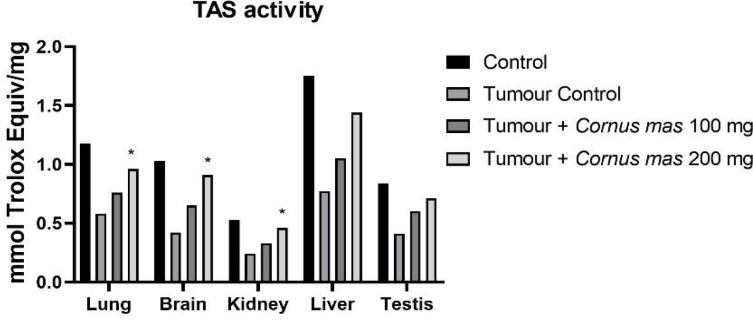
Mean TAS Activity Levels in All Groups. The data are expressed as mean ± standard deviation (*p<0.05 vs. Control group. One way ANOVA, post hoc Tukey test)

**Figure 8:**
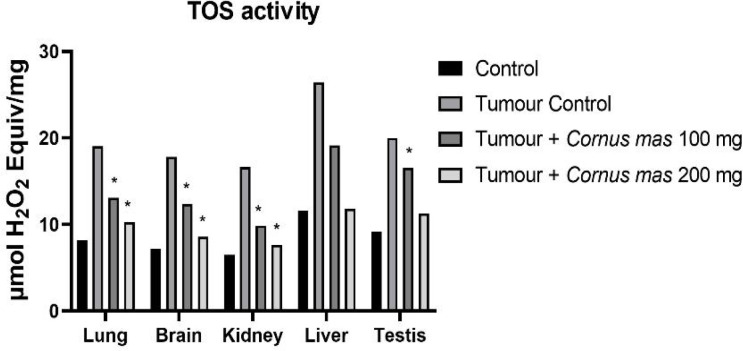
Mean TOS Activity Levels in All Groups. The data are expressed as mean ± standard deviation (*p<0.05 vs. Control group. One way ANOVA, post hoc Tukey test)

**Figure 9:**
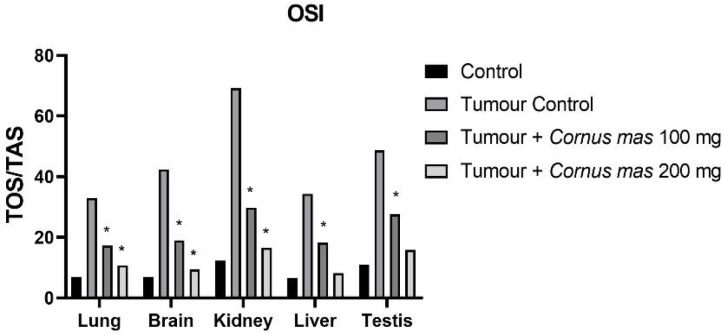
Mean OSI Levels in All Groups. The data are expressed as mean ± standard deviation (*p<0.05 vs. Control group. One way ANOVA, post hoc Tukey test)

The lowest GSH, SOD and TAS values were observed in the Tumor Control group in all tissues while the highest value was detected in the Control group among all tissues (Figure 4, Figure 6 and Figure 7 respectively). The other oxidative stress markers of GSSG, TOS and OSI showed adverse values. Thus, the lowest value for GSSG, TOS and OSI was detected in the Control group whereas the highest value was seen in the Tumor Control group (Figure 5, Figure 8 and Figure 9, respectively). Significant values for the GSH was seen in the Tumour + *C. mas* L 200 mg for lung, brain and kidney tissues. Also, in the Tumour + *C. mas* L 100 mg group, significant values were observed for the brain, kidney, liver and testis tissues (p<0.05). Regarding SOD activity in the lung, brain and kidney, the Tumor + *C. mas* L 100 mg group had a significant mean value and the Tumor + *C. mas* L 200 mg group in brain and kidney tissue but no significant value was seen in liver and testis tissues. Mean TAS activity values were observed in lung, brain and kidney tissues in Tumor + *C. mas* L 200 mg.

The OSI, GSSG and TOS levels are a common indicator for measuring oxident induced free radicals. The results showed that a statistically significant (p<0.05) increase for the OSI, GSSG, and TOS levels in tumerous tissues of mice when compared to the control groups. In contrast, the administration of *C. mas* L. fruit in a dose-dependent way showed significant reduction (p<0.05) in the OSI, GSSG and TOS levels in all of the tissues when compared to the tumor group (Figure 5, Figure 8, Figure 9. respectively).

## Discussion

Cancer is a disease that is characterized by an uncontrolled proliferation of cells forming tumors and rapidly spreading, resulting in death due to the proliferation of mostly surrounding tissues (Merlo et al., 2006). Some plants have been shown to be useful in the treatment of cancer. Plants, and vegetables used by people in various countries have led to the progression of cancer chemotherapy (Raju et al., 2012). It has shown that natural chemicals (such as resveratrol in red grapes, genistein and *C. Mas* L) that contain some of these plants have anti-cancer properties (Scartezzini et al., 2000). 

The cell lines obtained from cancer models and tumors are frequently used in studies on cancer. One of these models is the EAT model. EAT is one of the experimental animal tumors, but it has been subject to much research. (Nisari et al., 201; Taşkın et al., 2002). Yılmaz et al., (2019) in their study showed the antioxidant properties of the EAT tumor cells on the liquid and solid tumor models and reported that curcumin induced apoptosis. Ueki et al., (2013) investigated the regulatory effect of rats with cisplatin-induced renal inflammation and in the group with cisplatin, which they also gave curcumin (100mg/kg), the concentration of serum TNF decreased by 30%. In the group only given curcumin (50mg/kg), they stated that the serum TNF level was determined as 10% and decreased renal function disorders.

In this study 100 and 200 mg/kg *C. mas* L. fruit extract was administered as the treatment dose to the groups in which EAT cells were induced.

Our results show that tumors affect the oxidant defense in mice. By investigating the effect of *C. mas* L. fruit extract in a dose-dependent way in different tissues, our results extend previous findings where *C. mas* L. fruit extract has been found to have antioxidant effects. 

The *C. mas* L. fruit extract is an anticancer drug that is widely used in the treatment of cancers. Nonetheless, cancer cells interacting with oxygen and ions lead to an increase in superoxide and another ROS (Jia-Fu et al., 2012; Koyuncu et al., 2019; Reinert et al., 2013). An increase in the tumor-related ROS production in the tissues causes various side effects due to its cytotoxic and genotoxic effects. As a result, research has been conducted on the protective effects of a series of antioxidant substances on it providing protection against cancer (Alavian et al., 2014; Akyüz et al., 2018; Jia-Fu et al., 2012; Reinert et al. 2013; Zarei et al. 2014). In this study, for the first time, to the best of our knowledge, an examination of the protective effect of *C. mas* L. fruit extract on tissues of oxidant and antioxidant parameters such as various enzyme activities, GSH, GSSG, TOS OSI, and TAS, which have been altered in relation to cancer in tissues of tumor induced Balb/C mice. In addition, the potential role of *C. mas* L. fruit extract was investigated in the prevention of cancer-related antioxidant system, such as the increase in GPx, SOD, TAS, and GSH in the antioxidant defense system. Our findings showed a significant reduction in GPx and catalase activities and an enhancement in all tissue values in the Tumor Control group, but which were reversed approximately in the control levels in the groups. These results indicate oxidative stress induced by EAT modeling, which can be protected by *C. mas* L. extract.

Recent studies showed the potential value of *C. mas *L. fruits as a significant source of phenolic compounds and anthocyanins with high antioxidant activity, which varied greatly among the genotypes (Boris et al., 2012). 

In conclusion, our results showed that the *C. mas *L. fruit extract caused a reduction in the level of CAT activity, TOS, OSI, and GSSG levels, which showed an increase in relation to cancer presence, furthermore, in a dose-dependent manner, caused a correction of the deterioration in the antioxidant system related to tumors in the mice tissues. Testis and brain tissues were the most affected organs and the *C. mas* L. fruit extract had the most beneficial effect on these organs as well. To the best of our knowledge, this is the first study that confirms the beneficial effects of *C. mas* L. fruit extract on brain and testicle tissues.
